# Purinergic Receptors in Adipose Tissue As Potential Targets in Metabolic Disorders

**DOI:** 10.3389/fphar.2017.00878

**Published:** 2017-11-24

**Authors:** Marco Tozzi, Ivana Novak

**Affiliations:** Section for Cell Biology and Physiology, Department of Biology, University of Copenhagen, Copenhagen, Denmark

**Keywords:** purinergic receptors, adenosine, ATP, adipocytes, obesity, type-2 diabetes, inflammation, adipose tissue

## Abstract

Extracellular nucleosides and nucleotides, such as adenosine and adenosine triphosphate (ATP), are involved in many physiological and pathological processes in adipose tissue (AT). It is becoming accepted that, in addition to the well-established sympathetic and hormonal system, purinergic receptors contribute significantly to regulation of adipocyte functions. Several receptor subtypes for both adenosine (P1) and ATP (P2X and P2Y) have been characterized in white adipocytes (WA) and brown adipocytes (BA). The effects mediated by adenosine and ATP on adipocytes are multiple and often differing, depending on specific receptors activated. Using a variety of agonists, antagonists and transgenic animals it has been demonstrated that adenosine and P2 receptors are involved in lipolysis, lipogenesis, adipokines secretion, glucose uptake, adipogenesis, cell proliferation, inflammation, and other processes. Given their central role in regulating many AT functions, purinergic receptors are considered potential therapeutic targets in different pathological conditions, such as obesity and type-2 diabetes. To achieve this goal, specific and potent P1 and P2 receptors activators and inhibitors are being developed and show promising results. However, more insight is needed into the function of P2 receptors in brown and beige adipocytes and their potential role in thermogenesis. This review aims at summarizing current knowledge on the patho-/physiological role of P1, P2X, and P2Y receptors in WA and BA and their potential exploitation for pharmacological intervention. Furthermore, we analyze impact of purinergic signaling in AT – in health and metabolic diseases.

## Introduction

Metabolic disorders, such as obesity, dyslipidemia, and hyperglycemia, are closely related to AT dysfunction, and indeed AT is considered one of the most promising therapeutic targets (Guilherme et al., 2008; Kusminski et al., 2016). AT is a metabolic and endocrine organ consisting mainly of adipocytes. In addition, AT contains other cell types collectively named SVF, which includes MSCs, preadipocytes, endothelial cells, fibroblasts, and a variety of immune cells such as macrophages and T regulatory cells. Mammals have two main types of AT: WAT comprises mainly of WA, which store excess energy as triglycerides; and BAT, characterized by mitochondria-rich adipocytes, which express UCP1 that enables dissipation of energy by production of heat. Within WAT, there are also UCP1-positive cells with thermogenic capacity called beige or brite (brown-in-white) adipocytes. Cold exposure or other specific factors can produce browning of WAT (Harms and Seale, 2013; Kim and Plutzky, 2016). WAT and BAT are innervated by the sympathetic nervous system that together with hormones and other factors regulates adipocyte function (Bulloch and Daly, 2014). Adipocytes are also regulated by nucleosides and nucleotides, such as adenosine and ATP.

Generally, ATP is released from sympathetic nerves (Burnstock, 2007) and this is also likely in AT (Gnad et al., 2014), though only sympathetic nerves innervating BAT were found to express the ATP transporter VNUT (Razzoli et al., 2016). Many other non-excitable cells, such as epithelial, glial, stromal, and immune cells release ATP in basal conditions (Corriden and Insel, 2010) and in response to various patho-/physiological stimuli (Junger, 2011; Novak, 2011; Lazarowski, 2012). Similarly, ATP could be released from adipocytes (Gnad et al., 2014; Adamson et al., 2015). The mechanism of ATP release is not extensively studied; so far one study suggests channel pannexin-1 (Adamson et al., 2015) and another vesicular-based mechanism (Razzoli et al., 2016). Other cells in AT could also release ATP, contributing to a purinergic cross-talk between different cells, but such signaling is yet to be explored. In the extracellular space ATP is hydrolyzed to adenosine by one or more ecto-nucleotidases belonging to four families: ecto-nucleoside triphosphate diphosphorylases (CD39 type), ecto-5′-nucleotidase (CD73 type), ecto-nucleotide pyrophosphatase/phosphodiesterases (NPP) and alkaline phosphatases (APs) (Zimmermann et al., 2012). Interestingly, one of the enzymes NPP2, also known as phospholipase D or autotaxin, is multifunctional and released by adipocytes, and up-regulated autotaxin expression correlates with obesity (Rancoule et al., 2014). Nevertheless, it is most often assumed that adipocytes can release adenosine, possibly through nucleoside transporters (Antonioli et al., 2008; Gnad et al., 2014).

Adenosine, ATP, and other nucleotides signal through purinergic receptors that operate in virtually all mammalian cells, and it can be disturbed in various diseases, including metabolic syndrome, and therefore is attractive for therapeutic targeting (Burnstock, 2013; Burnstock and Novak, 2013; Chen et al., 2013; Jacobson and Muller, 2016). Here, we summarize the current knowledge about (i) the expression and function of adenosine receptors (P1R) and P2 receptors (P2XR and P2YR) in white, brown, and possibly in beige adipocytes; (ii) their role in the onset and progression of metabolic disorders; and (iii) their potential as therapeutic targets. In some cases, it is difficult to dissect the contribution of purinergic signaling in adipocytes from the AT/whole body effects (Peleli and Carlstrom, 2017). This is because the SVF in AT and other organs express purinergic receptors, and various conditions (e.g., inflammation) and experimental set up (e.g., whole-body receptor KOs or systemic receptor modulators) will complicate interpretation of effects.

## Adenosine Receptors in Adipose Tissue

Adenosine accumulates extracellularly in response to metabolic stress, tissue injury, hypoxia and inflammation and concentrations can range from nanomolar to micromolar in physiological and pathophysiological conditions, respectively (Fredholm, 2014). Adenosine can bind to four different G-protein-coupled receptors A_1_, A_2A_, A_2B_, and A_3_. A_1_R and A_2A_R have high affinity for adenosine, while A_2B_R and A_3_R have relatively lower affinity. A_1_R and A_3_R are coupled to G_i/o_ proteins and their activation inhibits cAMP production and decreases PKA activity. A_2A_R and A_2B_R are coupled to G_s/olf_ proteins and stimulate cAMP production, thus activating PKA. In addition, some adenosine receptors also activate PLC, Ca^2+^ signaling and mitogen-activated protein kinases (Fredholm et al., 2011).

**FIGURE 1:**
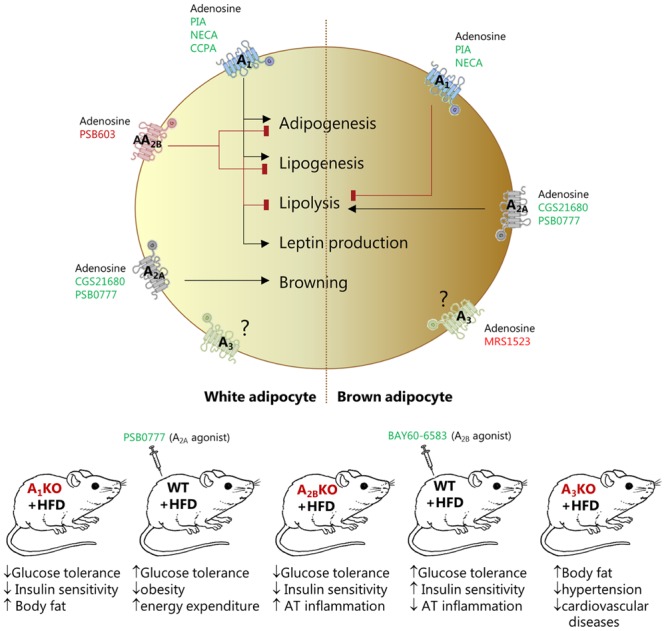
Effect of pharmacological and genetic modulation of adenosine receptors in white (left) and brown (right) adipocytes and in various mouse models. The figure shows agonists (green), antagonists (red) and knockout animals (red) that have been used in the studies quoted in this review to address the function of the specific receptor.

Adenosine has a key role in regulating many patho-/physiological processes in AT and adipocytes (**Figure 1**). The most studied adenosine receptor is the A_1,_ identified in WAT of many species (Trost and Schwabe, 1981; Larrouy et al., 1991; Strong et al., 1993; Mersmann et al., 1997; Tatsis-Kotsidis and Erlanger, 1999). Using pharmacological tools and whole body A_1_R KO mice, it was shown that activation of this receptor in rodents has anti-lipolytic effects mediated by inhibition of cAMP production and decrease in PKA and lipase activities (Fredholm, 1978; Schoelch et al., 2004; Dhalla et al., 2007a; Johansson et al., 2008). Rat WA are more responsive than BA to inhibition of lipolysis by the A_1_R stimulators PIA and NECA (Saggerson and Jamal, 1990), probably due to higher expression of the receptor in WA (Gnad et al., 2014). A_1_R activation also increases lipogenesis in mouse and rat WA (Johansson et al., 2008; Szkudelski et al., 2009). The A_1_R is also implicated in adipogenesis (Gharibi et al., 2012) and leptin production in WA (Cheng et al., 2000; Rice et al., 2000). All these A_1_R-mediated effects highlight the importance of adenosine signaling in AT, and predict impact on whole body metabolism. In accordance, A_1_R KO mice have increased fat mass and body weight, and impaired glucose tolerance and insulin sensitivity (Faulhaber-Walter et al., 2011; Yang et al., 2015). In contrast, mice overexpressing the A_1_R in AT are protected from obesity-induced insulin resistance (Dong et al., 2001).

Another fairly well studied receptor is the A_2B_R, detected in adipocytes and SVF (Gnad et al., 2014). *In vitro* experiments on murine pre-osteoblast cell line expressing either human A_2B_R or A_1_R showed that A_1_R stimulated adipogenesis, while the A_2B_R inhibited both adipogenesis and lipogenesis, supporting osteoblastogenesis pathways (Gharibi et al., 2012). In *in vivo* experiments, A_2B_R expression seems to correlate with parameters of obesity, both in rodents and humans, and the receptor is upregulated in visceral AT of mice fed HFD (Johnston-Cox et al., 2012). Genetic KO of the receptor is also associated with metabolic disorders. Whole body A_2B_R KO led to AT inflammation, insulin resistance, impaired glucose and lipid metabolism in mice (Johnston-Cox et al., 2012; Csoka et al., 2014; Peleli et al., 2015). Importantly, systemic administration of the A_2B_ specific agonist BAY 60-6583, following HFD regime, lowered plasma glucose, insulin and IL-6 levels, and ameliorated type-2 diabetes in mice (Johnston-Cox et al., 2012). These animal studies and a recent study (Johnston-Cox et al., 2014) are interpreted mainly in terms of the A_2B_R activation of WAT macrophage. Nevertheless, involvement of adipocyte A_2B_R cannot be excluded.

The A_2A_R may have a major role in BAT, where it is more abundantly expressed compared to WAT (Gnad et al., 2014). Early studies showed that adenosine inhibited lipolysis in BA from rat or hamster, probably via A_1_R (Schimmel and McCarthy, 1984; Woodward and Saggerson, 1986). In contrast, recent work shows that adenosine and A_2A_R agonists (CGS21680 or PSB-0777) activated lipolysis in human and murine BA, and to explain differences in the studies authors proposed species-related differential receptor expression (Gnad et al., 2014). Moreover, agonists used in this study increased energy expenditure, induced browning of WAT, improved glucose tolerance and protected C57Bl/6 mice from diet-induced obesity, thus revealing a promising thermogenic effect of adenosine. Similar effect on glucose homeostasis was reported for another A_2A_R agonist CGS21680 administered to Swiss strain mice fed with HFD (DeOliveira et al., 2017). No alteration in body weight or adiposity was detected, though decrease in some inflammatory markers was observed. The difference in animal obesity detected in the two studies could be due to different time regimes of drug treatment (8 weeks vs. 2 weeks), differences in strains of mice used (see below for comments on C57Bl/6 strain), or specificity of different A_2A_R agonists used.

There are only a few studies on A_3_R in adipocytes/AT. Isolated human WA express higher levels of A_3_R mRNA compared to BA. But inhibition of the receptor with MRS1523 had no significant effect on modulating lipolysis, at least in murine BA (Gnad et al., 2014). However, the A_3_R KO mice had less abdominal and total body fat, and mice were protected from hypertension and cardiovascular diseases in the chronic kidney disease model tested (Yang et al., 2016).

Taken together, there is strong evidence that adipocytes express all types of adenosine receptors that regulate patho-/physiological processes (**Figure 1**). There is a consensus that A_1_R regulates lipolysis and therefore FFAs levels, which play a significant role in the pathogenesis of insulin resistance, diabetes, and cardiovascular diseases (Dhalla et al., 2009; Antonioli et al., 2015). Several A_1_R agonists, e.g., SDZWAG994 (Ishikawa et al., 1998), ARA (Zannikos et al., 2001), and RPR749 (Shah et al., 2004), have been clinically evaluated as anti-lipolytic agents for the treatment of hypertriglyceridemia and type-2 diabetes. Though, development of full A_1_R agonists has been limited by (i) the debilitating side effects induced by the activation of the receptors in heart and kidney of animal models (Belardinelli et al., 1989; Wu et al., 2001); and (ii) a well-characterized desensitization of the receptor after repeated exposure to full agonists (Hoffman et al., 1986; Dhalla et al., 2007b). However, selective partial A_1_R agonists, e.g., CPA and GS-9667 (CVT-3619), effectively lowered plasma FFA levels without detectable cardiovascular side effects in rodents and humans (Dhalla et al., 2007a,b). These effects were achieved by administering lower concentrations of these drugs, which acted predominantly on AT, as it has larger A_1_R reserve compared to other tissues (i.e., atrioventricular node) (Staehr et al., 2013). Furthermore, given the role of the A_2B_R in glucose and lipid homeostasis, and AT inflammation, this receptor could be a promising target for the treatment of metabolic diseases. Finally, finding that activation of A_2A_R induces beiging of WA and activates BA (Gnad et al., 2014) may stimulate development of new pharmacological interventions for the treatment of obesity and metabolic disease.

## Purinergic P2 Receptors in Adipose Tissue

In contrast to adenosine, patho-/physiological functions of ATP and other nucleotides have not been studied so extensively in AT. Tri- and di-nucleotides signal through P2R belonging to two main families: the ionotropic P2XRs and the metabotropic G-protein coupled P2YRs. The P2XRs (P2X1–7) are ligand-gated cation channels activated primarily by ATP (North, 2016). The P2YR subtypes can be stimulated by different endogenous nucleotides and most potent ones (in humans) are given in brackets. P2Y1R (ADP), P2Y2R (UTP), P2Y4R (UTP), P2Y6R (UDP) receptors couple to G_q_ proteins and thus activate PLC-β, mobilizing Ca^2+^ from intracellular stores. P2Y11R (ATP) couple in addition to G_s_ proteins increase cAMP, while P2Y12R (ADP), P2Y13R (ADP), and P2Y14R (UDP, UDP-glucose) couple to G_i_ proteins and inhibit cAMP formation (von Kugelgen and Hoffmann, 2016).

**FIGURE 2:**
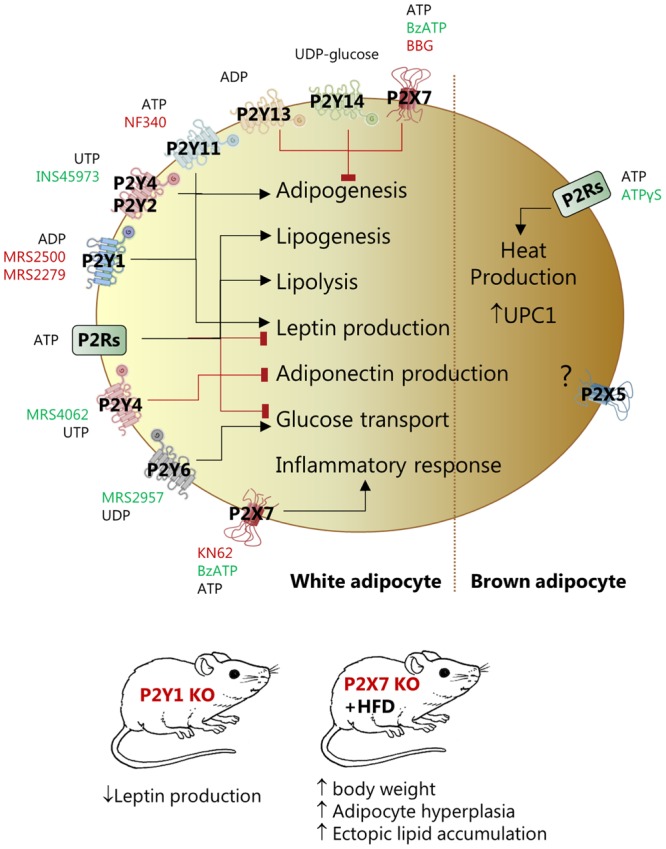
Effect of pharmacological and genetic modulation of P2X and P2Y receptors in white (left) and brown (right) adipocytes and in various mouse models. The figure lists agonists (green), antagonists (red) and knockout animals (red) that have been used in the studies quoted in this review to address the function of the specific receptor.

Early studies by Lee and Pappone (1997a,b) and Lee S.C. et al. (2005) used patch-clamp, Ca^2+^ imaging and mRNA analysis to fingerprint functional P2XRs and P2YRs in WA and BA. Here, we will focus on role of P2Rs in regulating multiple adipocyte-specific processes as summarized in **Figure 2**.

P2Rs regulate adipogenesis. Several studies used expression profiling and functional assays to describe that P2Y1R, P2Y2R or P2Y4R and P2Y11R positively affected adipogenic differentiation of stem cells derived from bone marrow or AT (Kawano et al., 2006; Zippel et al., 2012; Ciciarello et al., 2013; Li et al., 2016). In contrast, P2Y4R activation (UTP, MRS4062) inhibited cardiac AT-derived stem cells differentiation and P2Y4R KO mice developed bigger cardiac AT mass and higher expression of UCP1 (Lemaire et al., 2017). The opposite effect of P2Y4R in the mentioned studies could be related to the higher receptor expression in cardiac AT compared to other fat depots and/or beige/BA lineage in the cardiac tissue. Three other receptors have anti-adipogenic effects – P2Y13, P2Y14, and P2X7 (Zippel et al., 2012; Biver et al., 2013). Interestingly, P2X7R KO male mice had increased body weight, adipocyte hyperplasia in fat pads, and ectopic lipid accumulation in kidney, salivary glands, and pancreas (Beaucage et al., 2014). Other *in vitro* and *in vivo* studies indicate that the P2X7R stimulation directs differentiation of MSCs toward the osteoblast lineage rather than toward adipocytes (Li et al., 2015).

P2Rs are also implicated in lipid metabolism. One study on isolated rat WA shows that ATP, UTP, and BzATP, probably by activating different P2Rs, had dual effects – activated lipolysis and inhibited insulin-induced leptin production (Lee H. et al., 2005). In contrast, no lipolytic effect of ATP was found in a similar cellular model (Schodel et al., 2004). The explanation for these divergent results could be that the two studies used different ATP concentrations, thus affecting different palette of P2Rs. Furthermore, in the latter study on isolated WA, ATP stimulated lipogenesis but had no effect on glucose transport (Schodel et al., 2004).

The role of purinergic signaling in glucose uptake in adipocytes has been investigated in 1980s. Two studies showed that low concentrations of ATP inhibited insulin-stimulated glucose transport in rat fat cells (Chang and Cuatrecasas, 1974; Halperin et al., 1978), and it was assumed that extracellular ATP had direct inhibitory effect on the insulin receptor. More recently, it was shown that P2Y6R activation by UDP or MRS2957 increased GLUT-4 translocation and glucose uptake in primary WA and 3T3-L1 cells (Balasubramanian et al., 2014).

In addition to effects on adipogenesis, lipid metabolism, and glucose transport, P2Rs affect leptin and adiponectin production and secretion, but activation of different P2R subtypes might lead to opposite effects. ATP and BzATP reduced leptin mRNA levels and inhibited insulin-induced leptin secretion in rat WA (Lee H. et al., 2005). In another study, inhibition of P2Y1R by MRS2500 decreased leptin production under basal and insulin-stimulated conditions in isolated mouse WA (Laplante et al., 2010). Furthermore, the study showed that plasma leptin was lower in mice lacking P2Y1R, however, in mice on HFD the plasma leptin was enhanced and the inhibitory effect of receptor KO was not observed. Stimulation of P2Y4R by UTP or MRS4062 inhibited adiponectin expression and secrection in cardiac adipocytes and P2Y4R KO mice showed increased adiponectin secrection in hypoxia and a cardioprotective phenotype (Lemaire et al., 2017).

Adenosine triphosphate is generally considered as an inflammatory molecule (Idzko et al., 2014). Particularly the P2X7R mediates inflammation in AT. In primary adipocytes from rat epididymal fat, millimolar concentrations of ATP evoked inflammatory response and led to impaired insulin signaling and glucose uptake (Yu and Jin, 2010). Visceral and subcutaneous human AT express functional P2X7R, which could be involved in release of inflammatory cytokines such as IL-6, TNFα, and PAI-1 (Madec et al., 2011). Interestingly, this study showed that P2X7R expression appeared to be high in adipocytes isolated from subjects affected by metabolic syndrome. The P2X7R and NLRP3 inflammasome expression and IL-1β secretion was elevated in metabolically unhealthy obese individuals and the receptor expression correlated with body mass index and metabolic syndrome scores (Pandolfi et al., 2015). Whether this was due to effects on adipocytes or infiltrating immune cells is not clear (Pandolfi et al., 2016). In contrast, an earlier study concluded that the P2X7R was not involved in obesity-associated inflammasome activation. This was based on the observation that P2X7R KO mice on the C57BL/6 background and fed on HFD were not protected from obesity, AT inflammation and associated metabolic abnormalities (Sun et al., 2012). The C57BL/6 mice though have a single nucleotide polymorphism in the P2X7R that compromises the immune response (Rissiek et al., 2015), which could explain discrepancies in inflammasome activation in the two studies.

Rodent BA, express several P2X and P2Y receptors and stimulation with ATP leads to exocytosis and heat production (Lee and Pappone, 1997a; Lee S.C. et al., 2005). In a more recent study, ATPγS, enhanced UCP1 expression and induced browning in BAT in conditions of low adaptive thermogenesis and b-adrenergic receptor KO mice (Razzoli et al., 2016). This effect is most likely exerted via more than one receptor and P2X5R, P2X7R, and P2Y12R are overexpressed in β-less BAT. Interestingly, P2X5R is proposed as a novel cell surface marker for beige and BA as its mRNA levels are markedly higher in mouse BAT compared to WAT and other tissues (Ussar et al., 2014). Furthermore, the P2X5R expression increased in both BAT and subcutaneous WAT upon chronic cold exposure, paralleling expression of UCP1 (Ussar et al., 2014; Garcia et al., 2016; Razzoli et al., 2016). However, mechanisms of P2X5R mediated effects in AT are unknown.

Taken together, there is good evidence that P2Rs affect a wide range of patho-/physiological processes in rodent and human AT. Many of these processes, e.g., lipid deposition, metabolism, endocrine activity, and inflammation, are deregulated during pathological states such as obesity and diabetes. However, P2Rs modulators have not yet been tested in clinical trials for treatment of metabolic disorders. Future research is still needed to dissect functions of P2R subtypes in adipocytes and AT before best P2R targets and drugs are selected.

## Conclusion and Perspectives

In this review, we discussed contribution of P1 and P2 receptors to modulation of AT functions and considered processes that may underlie their role in metabolic disorders. Several preclinical studies indicate that pharmacological manipulation of purinergic signaling in adipocytes and AT has interesting potential for treating metabolic disorders. However, translation of these findings into clinical trials will require more detailed knowledge about the role of extracellular ATP and adenosine in the onset and progression of obesity-related disorders, as well as about the basic physiology and pharmacology of purinergic receptors expressed in adipocytes and in AT. In this context, it will be necessary to: (i) know differential expression of adenosine and P2Rs in white, brown and beige adipocytes belonging to different fat depots (subcutaneous, visceral, cardiac etc.); (ii) clarify sources and concentrations of nucleotides/sides and modifying enzymes present in specific AT microenvironments; (iii) understand role of purinergic system in interplay between different cells in AT microenvironment and potential patho-/physiological conditions which may affect those.

## Author Contributions

MT wrote the draft and prepared figures. IN contributed to planning and writing of the review.

## Conflict of Interest Statement

The authors declare that the research was conducted in the absence of any commercial or financial relationships that could be construed as a potential conflict of interest. The reviewer GY declared a past co-authorship with one of the authors IN to the handling Editor.

## Abbreviations

AT: adipose tissue
ADP: adenosine diphosphate
ATP: adenosine triphosphate
BA: brown adipocytes
BAT: brown adipose tissue
BzATP: 2′-3′-*O*-(4-benzoylbenzoyl)-ATP
cAMP: cyclic AMP; FFAs, free fatty acids
HFD: high fat diet
IL: interleukin
KO: knockout
MSCs: mesenchymal stem cells
PAI-1: plasminogen activator inhibitor-1
PKA: protein kinase A
PLC: phospholipase C
SVF: stromal vascular fraction
TNFα: tumor necrosis factor-α
UCP1: uncoupling protein-1
UDP: uridine diphosphate
UTP: uridine triphosphate
VNUT: vesicular nucleotide transporter
WA: white adipocytes
WAT: white adipose tissue
